# The Case for Co-production in Singapore's Mental Healthcare

**DOI:** 10.3389/fpsyt.2021.740391

**Published:** 2021-11-17

**Authors:** Ying Ying Lee, Suying Ang, Charmaine Tang

**Affiliations:** ^1^Research Division, Institute of Mental Health, Singapore, Singapore; ^2^Early Psychosis Intervention Programme, Institute of Mental Health, Singapore, Singapore

**Keywords:** psychosis, co-production, psychosocial treatment, participatory, collaboration

## Abstract

For better or worse, there exists a power differential between psychiatrists and their patients in mental healthcare. Co-production was proposed to be the “third space” to offer truce between the professional-patient tension in mental healthcare. In Singapore, co-production is a new, but growing, approach to mental healthcare service delivery. In this commentary, we argue that co-production is not just a novel way to provide service, but a moral imperative. Recovery Colleges and its adoption in Singapore is discussed in some detail to highlight how co-production may be applied in practice.

## Introduction

I was inducted to the mental healthcare system under unfortunate circumstances in 2013. My family sought help for me at a tertiary psychiatric hospital because I was experiencing symptoms of paranoia, delusions and disorganized thoughts and behaviors. Eventually, I was diagnosed with schizophrenia. Experiencing psychosis took away my autonomy and rights as a human being in some ways. One of which was the loss of my mental capacity to seek medical treatment. For better or for worse, there exists a power differential between psychiatrists and their patients in mental healthcare. Psychiatrists wield a certain power over their patients as healthcare providers by having the power to legally mandate treatment on their patients. For instance, psychiatry is the only medical specialty that is empowered by the law to mandate treatment on its patients (1, 2). Even though the decision to invoke the Mental Health (Care and Treatment) Act for involuntary admission is based on a thorough assessment and criteria, treatment can include enforced hospital admissions, injections of sedative and/or psychiatric medications, and in some cases, physical and/or chemical restraints. Of course, I would like to believe that all psychiatrists exercise such power with care, and only in some cases. In Singapore, doctors are held in great esteem and they enjoy a high social status as members of a highly regarded profession (3). As a result, local patients very often treat their doctor's advice and instructions with great reverence. Hence, even when a patient seeking psychiatric help is in remission, such a power differential can loom over—unseen, unheard, subtly influencing the doctor-patient dynamics.

Besides gaining medical remission and functioning, my journey of recovery included rebuilding my sense of self and autonomy as an active citizen of my community. I had an excellent care team, which included case managers and psychiatrists, who always took my values and goals into consideration when providing care for me. In fact, we got along so well that I got invited to join their multidisciplinary team as a peer support specialist. After a 2-year stint with them, I joined Institute of Mental Health's (IMH) research department. Despite the power differential between psychiatrists and their patients, there are efforts to level the playing field. In the landmark Salzburg Statement on Shared Decision Making (2011), a group of professionals and patients agreed on a statement that called on both patients and clinicians to be co-producers of health. It described a shifting boundary between expert and non-expert, where patients were called on to play a more active role in the decisions regarding their healthcare needs and increasing involvement of patients and the public as key stakeholders in public healthcare. In the same vein, the IMH's senior management, in collaboration with its staff, has come up with seven aspirations to shape the future of local mental healthcare services. One of them is to collaborate and co-create with patients, caregivers, and partners for care delivery. By leveraging the unique experience of patients, caregivers, and partners to bolster current services, we hope to develop more person-centric and innovative care models. As this initiative only started in 2021, the champions of the co-creation workgroup has been working with our senior leaders and Corporate Development team to identify best practices for the development of new care models. It helps to address the power differential between healthcare providers and patients. Benefits of addressing the issues of power between healthcare provider and patients include promotion of shared decision-making during consultations. Shared decision-making has been linked with better health outcomes (4), higher patient satisfaction, and patient adherence to treatment (5).

One of the latest offerings in the mental healthcare landscape beyond our shores is co-production. Co-production and co-creation are often used interchangeably when referring to service user involvement in the development and delivery of public services. However, it was suggested that co-production and co-creation are distinct processes in the cycle of service user involvement (6). Co-creation was defined as user involvement at the strategic level, during the planning and designing of services, while co-production was defined as user involvement at the implementation level, during the delivery of services (6). The integration of peer support services within IMH is an effort to promote more co-production in mental healthcare delivery locally (7).

## What is Co-production?

Co-production has been heralded as the “third space” to offer truce between the professional-patient tension in mental healthcare (2). This term was first coined by Nobel laureate Elinor Ostrom et al., and later operationalized by law professor Edgar Cahn. First introduced to solve the skyrocketing youth crime rates during the 1970s in the Chicago area, this concept has evolved into a key working principle in the UK's mental healthcare system (8). Today, a working definition of co-production is “delivering public services in an equal and reciprocal relationship between professionals, people using services, their families and their neighbors” (8). Co-production fosters reciprocal relationships between mental healthcare professionals, the recipients of their services, and others in the social network via collaborations and partnerships. There is an element of power and knowledge sharing between professionals and the layperson stakeholders. A useful way to describe co-production is to draw upon Arnstein's Ladder of Participation (9). At the lowest level, there are services that are “doing to” recipients of care, where recipients are not given a choice. In the second level, there are services that are “doing for” recipients of care, where recipients are engaged in care. In the third level, there are services that are “doing with,” where recipients are co-designing and co-delivering services (10).

There are four main tenets in co-production, as advocated by Cahn (11). Firstly, it is to recognize people as assets because people themselves are the true wealth of the society. Secondly, it is to recognize that work and productivity may look different for different people. For example, a mental health advocate, who may not be holding a full-time job, “work” may not always be directly translated into economic gains. Thirdly, promoting reciprocity by giving and receiving, because it builds trust between people and fosters mutual respect. Fourthly, it is building social networks because people's physical and mental well-being depends on strong enduring relationships. We would argue that co-production is not just a novel way of producing and delivering mental healthcare services, it is a moral imperative. The challenges posed by mental health issues are quite unlike other physical illnesses because of its unique socioeconomic consequences. Persons in recovery from mental health issues in Singapore face tremendous stigma and discrimination from the public and the workplace (12, 13). These studies on the level of stigma against persons with mental health issues in Singapore consistently suggest that they were excluded from social circles and denied work opportunities because of their conditions. As a result, the quality of life for this group of people suffers, possibly due to the systemic social and occupational discrimination (14). Co-production can be an approach where the knowledge of lived experiences can be harnessed to co-create a more humane and empathetic mental healthcare system in Singapore. The marriage between the expertise of professionals and experiences of patients could provide medically informed services that are sensitive to the struggles of its recipients.

## Co-production in Action: The Emergence of Recovery Colleges

Recovery Colleges are emerging as platforms for co-production in mental healthcare worldwide (15, 16). The idea was first mooted in the US, and then manualized in the UK (15). Since the emergence of the first Recovery College in London in 2008, it has spread to regions like Australia, Hong Kong, and Singapore (16). Recovery Colleges provide an alternative form of intervention for the recovery of persons with mental illness. Defining features of a Recovery College included, but are not limited to, (1) a focus on co-production, where classes are co-produced and co-delivered by professionals and persons in recovery, (2) open for everyone in the mental health community, to promote interactions between mental health professionals and persons in recovery on a level playing field, and (3) operates on college principles, where students register for classes, plan their own timetables, and classes are educational in nature (15). Modeling after UK's Recovery College, four non-profit organizations, with support from Jardine Matheson Group MINDSET, came together to establish the Mindset College in Hong Kong. With co-production as its bedrock value and mode of operation, they have created educational courses for persons in recovery from mental health issues (17).

Although co-production is a growing trend in mental healthcare service designing and delivery in the UK (18, 19), there is a dearth of evidence base from Singapore. To the authors' best knowledge, only one paper was published by two local authors based in Singapore on the theoretical principles of collaborations, co-productions and network (20). From a public policy perspective, they delved into the definitions of collaboration, co-production and created networks based on four case studies in the local context. The authors (20) cautioned that co-production in its local context may lose its original intent of encouraging active citizen participation to become a form of cost-efficient volunteer management model. In Hong Kong, recovery in mental healthcare is a burgeoning field. A small but vocal group of mental health professionals are rallying service users to be involved in their care, and are advocating on their behalf on the international stage for the value and challenge of co-production in an Asian society (21–23).

Recovery Colleges in Singapore is in its infancy now. Resilience Collective, a local social service agency, was set up in 2018 to emulate the Recovery Colleges model in the UK (24). Co-managed by staff who are persons in recovery, Resilience Collective co-produced workshops on topics that explores recovery from mental illness, managing anxiety, art of friendship, and managing self-stigma to support members in the mental health community. School of Ability and Recovery (SOAR) was started in 2018 as a ground up initiative modeled after Recovery Colleges. With the support of Youth Corps Singapore and like-minded volunteers, SOAR ran two workshop series in 2019, totaling to 10 workshops on topics like mindfulness, self-care, what is psychosis and dealing with workplace stigma (25).

A challenge faced when sourcing for funding for Recovery Colleges in Singapore is the lack of evidence to support the efficacy of such co-production efforts on patient outcomes locally. Even though quantitative and qualitative evidence for co-production from Europe is growing (26, 27), the import of new ideas across continents is sometimes met with a level of skepticism in Singapore, especially of its transferability to the local context and culture. However, the lessons learnt by our European colleagues may serve as foresight for us. For instance, when conducting co-production trainings, considering into its long-term efficacy is crucial. Continuous training and long-term supervision may benefit practitioners of co-production (26). Moreover, due to the flexible nature of co-production, the context in which co-production is implemented must be considered when one performs evaluation projects on co-production (27).

As frustrating as the power differential between psychiatrists and patients can be for some, change is gradually making its way into the mental healthcare system. Efforts to co-create a collaborative mental healthcare landscape is currently underway. In fact, the National Healthcare Group has approved a pilot grant to evaluate the outcomes of co-producing and co-delivering educational workshops for persons with first episode psychosis in November 2020. Various stakeholders, like patients, caregivers, members of public and mental health professionals, were invited to join sessions to brainstorm and co-create educational workshops. To date, we have run 9 hours of co-produced online workshops (called *Striking Matches*) in collaboration with the Early Psychosis Intervention Programme in Singapore. Preliminary results indicate that there was a modest increase in mental well-being, personal recovery, and social inclusion in participants with first episode psychosis. This project is the first of its kind in Singapore. A long-term goal of this project is to build the evidence base for co-production in the local mental health community.

In the UK, the presence of Recovery Colleges impacts more than just patients in recovery from mental health issues, it was also reported that they change the way staff and society view persons in recovery (28). As a result, the mental health services in the UK changed in a fundamental way. No longer are patients viewed as disabled, passive recipients of services, but they are enabled, active members making contributions to the mental health community. We believe that there is something for Singapore's mental healthcare system to learn from our English counterparts. Co-production may be part of the solution in the recovery of our mental healthcare system.

## Conclusion

Experiencing psychosis can be dehumanizing in some ways. The existing power differential between psychiatrists and patients does not always help to promote recovery and shared decision-making in mental healthcare services. Co-production, an emerging approach to mental healthcare, may be a candidate to address issues brought about by power differential in mental healthcare services. Recovery College is a novel approach to mental healthcare intervention that is an epitome of co-production. While Recovery Colleges are common in the UK, it is still on its way to be fully supported by healthcare professionals, funders and policymakers in Singapore. Yet, there is a growing number of programs and services adopting co-production in its workflow locally. In Singapore, there is Resilience Collective and the SOAR initiative. More recently, there is also a funded pilot project on co-producing workshops for persons with psychosis going on at the IMH since March 2021. Seeing how co-production has changed the UK mental health services in fundamental ways, some practitioners in Singapore are holding the hope that this approach to mental healthcare can transform our mental healthcare services to a more empowering one for our patients.

## Data Availability Statement

The original contributions presented in the study are included in the article/supplementary material, further inquiries can be directed to the corresponding author.

## Ethics Statement

Written informed consent was obtained from the individual(s) for the publication of any potentially identifiable images or data included in this article.

## Author Contributions

YYL conceptualized and wrote the first draft of the paper. SA and CT gave intellectual feedback to the manuscript based on their clinical and professional expertise. All authors contributed to the article and approved the submitted version.

## Funding

This work was partially supported by a Population Health Grant from the National Healthcare Group (PHG20.S.I.2.7).

## Conflict of Interest

The authors declare that the research was conducted in the absence of any commercial or financial relationships that could be construed as a potential conflict of interest.

## Publisher's Note

All claims expressed in this article are solely those of the authors and do not necessarily represent those of their affiliated organizations, or those of the publisher, the editors and the reviewers. Any product that may be evaluated in this article, or claim that may be made by its manufacturer, is not guaranteed or endorsed by the publisher.
